# Retinal pigment epithelium hyperplasia overlying pigment epithelial detachment in age-related macular degeneration can masquerade as neovascularization on optical coherence tomography angiography

**DOI:** 10.1007/s00417-018-4138-y

**Published:** 2018-09-18

**Authors:** Ling Chen, Xiongze Zhang, Yuhong Gan, Bing Liu, Yuxin Zhang, Feng Wen

**Affiliations:** State Key Laboratory of Ophthalmology, Zhongshan Ophthalmic Center, Sun Yat-sen University, 54 Xianlie Road, Guangzhou, 510060 China

**Keywords:** Age-related macular degeneration, Pigment epithelial detachment, Retinal pigment epithelium hyperplasia, Optical coherence tomography angiography, Image artifacts

## Abstract

**Purpose:**

To report the image artifacts due to retinal pigment epithelium (RPE) hyperplasia overlying retinal pigment epithelial detachment (PED) in age-related macular degeneration (AMD), which can masquerade as neovascularization on optical coherence tomography angiography (OCTA).

**Methods:**

A hospital-based, retrospective, and cross-sectional study. Twenty-two eyes from 16 patients with non-vascularized PED related to AMD were included in this study. All patients were examined by OCTA, spectral-domain optical coherence tomography, fluorescence angiography, and indocyanine green angiography. Vascular flow signals (VFS) on both the outer retinal slab of en face OCTA and cross-sectional OCTA and their correspondence with RPE hyperplasia were evaluated.

**Results:**

Fifteen eyes (68.2%) showed VFS on both the outer retina slab of en face OCTA and cross-sectional OCTA, all corresponding to the RPE hyperplasia overlying PED. Among them, 12 eyes with lump RPE hyperplasia outside foveal avascular zone (FAZ) all showed obvious VFS on the outer retina slab of OCTA, and 3 eyes with scattered RPE hyperplasia outside FAZ showed VFS fragments. Of note, 4 eyes had accompanied RPE hyperplasia inside FAZ, and 7 eyes without RPE hyperplasia overlying PED showed no corresponding VFS on the outer retina slab of OCTA. Additionally, a round-like dark band at the edge of PED was observed in the outer retina slab on en face OCTA in 17 eyes (77.3%).

**Conclusions:**

RPE hyperplasia overlying PED in AMD can masquerade as neovascularization on OCTA. To avoid misdiagnosis and unnecessary treatment, this RPE hyperplasia-related image artifact should be considered when interpreting OCTA images.

## Introduction

Optical coherence tomography angiography (OCTA) is a non-invasive technology that uses motion contrast to produce high-resolution angiographic images of the retinal and choroidal vasculature, without the need for a contrast agent [1, 2]. OCTA has been shown to be valuable in the detection and evaluation of vascular abnormalities in different retinal and choroidal diseases including diabetic retinopathy (DR), retinal vascular occlusion (RVO), macular telangiectasia, choroidal neovascularization (CNV) due to age-related macular degeneration (AMD), and central serous chorioretinopathy (CSC) [3–7]. Its use is rapidly expanding in clinical practice as well as in research of the pathophysiology of retinal and choroidal diseases. However, recent studies have described different types of image artifacts in OCTA, which are very common in OCTA of the retina and choroid and more frequent in eyes with pathology [8–11].

Retinal pigment epithelial detachment (PED), which is defined as the anatomical separation of the retinal pigment epithelium (RPE) from underlying Bruch’s membrane, might be associated with various retinal disorders [12]. The most common disease associated with PED is AMD. The classification of PED in AMD can be divided into drusenoid, serous, vascularized, or mixed categories [12]. As we know, traditional imaging techniques, including fluorescence angiography (FA), indocyanine green angiography (ICGA), and spectral-domain optical coherence tomography (OCT), can be used to identify PED types and determine whether neovascularization exist. OCTA, as a non-invasive technology, give us further information, including cross sectional and multiple en face layers of retina and choroid. A recent study demonstrated that OCTA had a sensitivity of 76% and specificity of 61% for detecting vascularized PEDs [13]. OCTA have been more and more used to detect whether there is neovascularization in patients with AMD and to explore the potential factors of PED formation. However, in our clinical practice, it was not uncommon that non-vascularized PED was misdiagnosed as type 3 neovascularization secondary to AMD. Since a mixed serous and drusenoid PED with a closely related deep neovascular lesion is typical characteristic of type 3 neovascularization [14].

Recent studies have reported the image artifacts in OCTA caused by PED or soft drusen, mainly including masking and unmasking artifacts [8, 15]. However, there is another type of artifacts caused by RPE hyperplasia overlying PED, which has not been reported before. To the best of our knowledge, the RPE hyperplasia-related artifacts are very common in patients with PED related to AMD and cannot be removed by the available artifact removal algorithm. The RPE hyperplasia-related artifacts might have important clinical significance for it can masquerade as neovascularization in both the outer retinal slab of en face OCTA and the cross-sectional OCTA. Failure to properly recognize this image artifact might lead to incorrect diagnosis and management of the disease.

The purpose of the present study is to report the image artifacts caused by RPE hyperplasia overlying PED in AMD, which can masquerade as neovascularization on OCTA. Of note, we only focused on the image artifacts of vascular flow signals (VFS) at the outer retinal slab of OCTA in patients with PED related to AMD in this study. Since under normal conditions, there is no VFS at the outer retinal slab of OCTA, which is very important for judging neovascularization.

## Materials and methods

This was a hospital-based, retrospective, and cross-sectional study of patients with non-vascularized PED related to AMD who underwent OCTA, OCT, color fundus photography (FP), autofluorescence (AF), FA, and ICGA examination in the Zhongshan Ophthalmic Center between July 2017 and February 2018. The research adhered to the tenets of the Declaration of Helsinki and was approved by the Institutional Review Board of the Zhongshan Ophthalmic Center at Sun Yat-sen University. Potential risks associated with the ICGA and FFA examination were fully discussed with the patients, and a written informed consent on invasive examination was obtained from all included patients.

## Patients

The inclusion criteria were patients aged more than 50 years with the presence of unilateral or bilateral non-vascularized PED secondary to AMD, including drusenoid PED [16], serous PED, or mixed PED with a drusenoid and serous components. FA, ICGA, and OCT were used to classify PEDs as non-vascularized or vascularized. The exclusion criteria included presence of any other retinal disease such as neovascular AMD, polypoidal choroidal vasculopathy (PCV), and central serous chorioretinopathy (CSC) based on FA and ICGA, or any systemic conditions associated with PEDs. Moreover, patients who had a previous ophthalmological intervention procedure, such as laser coagulation, vitrectomy, anti-vascular endothelial growth factor injection, or photodynamic therapy were excluded from this study. Patients with evidence of significant cataracts or vitreous opacity, which influence imaging quality, were also excluded from the study.

## Data collection

Demographic information, medical records, and multimodal imaging data were collected. The best-corrected visual acuity (BCVA) was measured with Snellen charts; color FP was performed with a Zeiss FF450 Plus fundus camera (Carl Zeiss, Inc., Jena, Germany). ICGA, FFA, and AF were performed with Heidelberg retina angiogram (Spectralis HRA, Heidelberg Engineering, Heidelberg, Germany). Spectral-domain optical coherence tomography (SD-OCT) was performed with an HRA + OCT Spectralis (Heidelberg Engineering, Heidelberg, Germany).

## Optical coherence tomography angiography

OCTA images were obtained using the AngioVue imaging system (Optovue Inc., CA, US), with split-spectrum amplitude-decorrelation angiography (SSADA) software [17], which can visualize the retinal and choroidal vasculature and cross section simultaneously. This system has a scan speed of 70,000 A-scans per second and uses a light source of 840 nm with bandwidth of 45 nm. Each OCTA volume contained 304 × 304 A-scans, with two consecutive B-scans captured at each sampling location. Two orthogonal OCTA volumes were acquired to minimize motion artifacts. Active eye tracking system was available to reduce noise and motion artifacts. All eyes were scanned using a 6 × 6-mm protocol, centered on the fovea.

The OCTA scans were automatically segmented by the software into four “en face” OCT slabs: (1) superficial retinal slab (SRL) from 3 μm beneath the internal limiting membrane to the 15 μm beneath the interface of the inner plexiform layer and inner nuclear layer (IPL/INL), (2) deep retinal slab (DRL) from the 15 μm beneath the IPL/INL to 71 μm beneath the IPL/INL, (3) outer retinal slab from 71 μm beneath the IPL/INL to 31 μm beneath the RPE, and (4) choriocapillaris from 31 μm beneath the RPE to 61 μm beneath the RPE. Furthermore, an artifact removal algorithm is available for the outer retinal slab and used to automatically remove projection artifacts related to overlying retinal vessels.

## Image evaluation

Two retina physicians (LC and XZZ) independently assessed the OCTA images on the instrument software (XR Avanti system 2016.2.0.35), as well as other multimodal images. We performed a dynamic OCTA volume analysis. All OCTA B-scans were evaluated to ensure accurate segmentation of the layers for all four slabs, with manual corrections of the segmentation lines when required. En face OCTA images were assessed whether VFS existed in the outer retinal slab. Then we examined the cross-sectional OCTA to determine the origin of the VFS shown in the outer retinal slab. Furthermore, multimodal images were evaluated to explore the cause of VFS on OCTA. Analysis of the foveal and parafoveal abnormal flow was performed. To accurately position the foveal center, we used the SRL OCTA scan as a benchmark (Fig. 3e). A 0.5-mm circle was drawn in the central foveal avascular zone (SRL-FAZ). Then a corresponding circle was drawn in the outer retinal slab of en face OCTA (Fig. 3f), as well as in color FP, infrared (IR) FP, FA, and ICGA images (Fig. 3a–d) [18]. Discrepancies between the two ophthalmologists were resolved by consulting a retina specialist (FW).

## Statistics

Statistical analyses were performed with SPSS Version 21.0 software (SPSS, Inc., Chicago, IL). Descriptive statistics (means ± standard deviations [SDs]) of normally distributed variables (age) were calculated.

## Results

A total of 22 eyes from 16 patients with non-vascularized PED related to AMD were eligible for inclusion in this study. The ages ranged from 53 to 82 years, with a mean age of 68.6 ± 7.5 years, and the male-female ratio was 3:1.

Of the 22 eyes with non-vascularized PED related to AMD, 15 eyes (68.2%) showed VFS at the outer retina slab of en face OCTA and cross-sectional OCTA. The position and pattern of the VFS displayed in the outer retina slab on en face OCTA were corresponding to the position and pattern of the RPE hyperplasia overlying PED, which was confirmed by other multimodal imaging including color FP, AF, FA, ICGA, IR, and OCT.

**Fig. 1:**
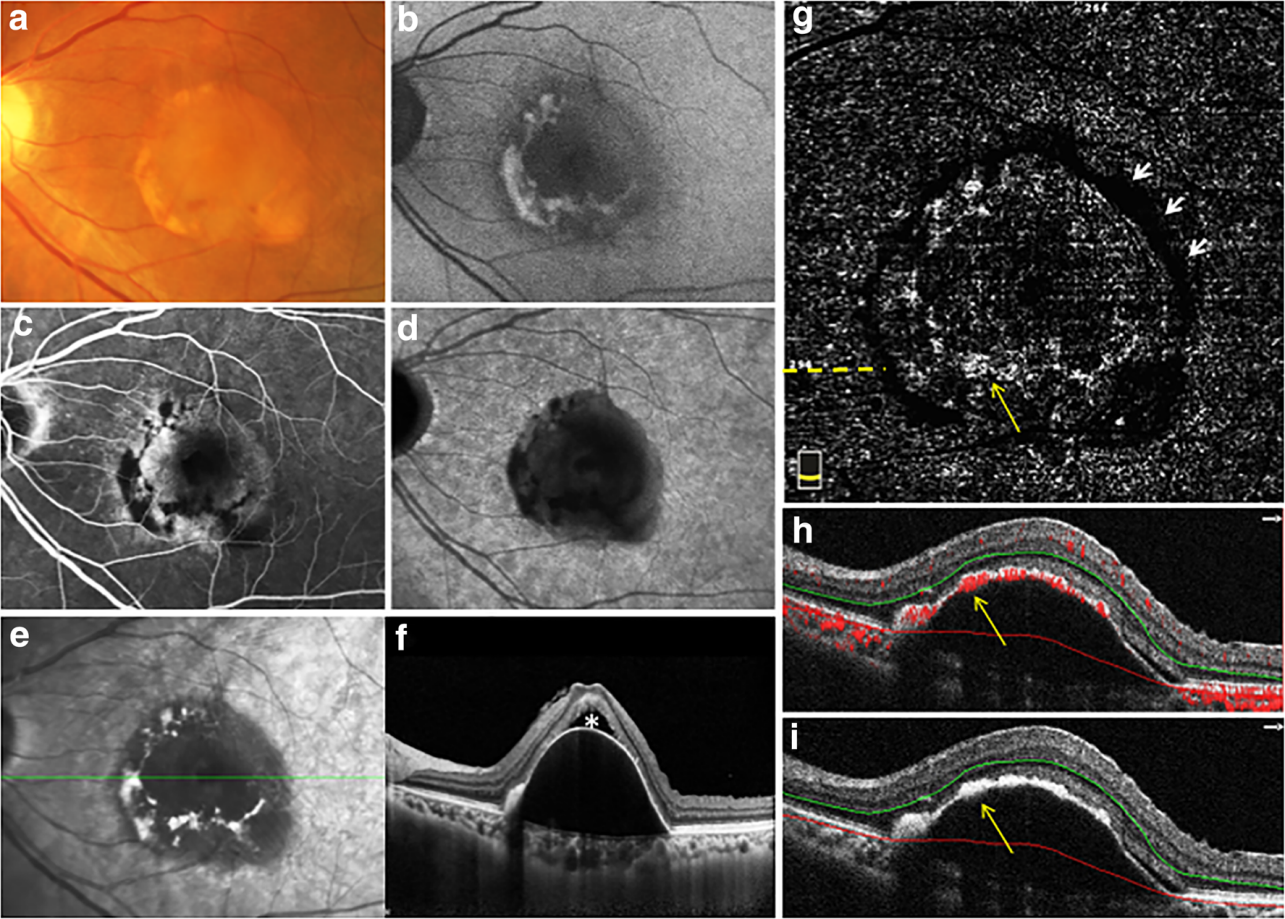
Multimodal imaging of non-vascularized PED in AMD with lump RPE hyperplasia outside the central foveal avascular zone. A non-vascularized PED imaged on color fundus photography (**a**), fundus autofluorescence (**b**), late-phase fundus fluorescein angiography (**c**), late-phase indocyanine green angiography (**d**), infrared fundus photography (**e**), spectral-domain optical coherence tomography (**f**), the outer retina slab on en face OCTA (**g**), cross-sectional OCTA, and cross-sectional OCT with segmentation boundary lines (**h** and **i**). The solid line in **e** shows the location of B-scans in **f**. The asterisk shows the sub-retinal fluid. The dotted line in **g** shows the location of B-scans in **h** and **i**. The long yellow arrow shows the abnormal flow corresponding to the RPE hyperplasia overlying PED at the same location. The short white arrows show the round-like dark band around the edge of PED

**Fig. 2:**
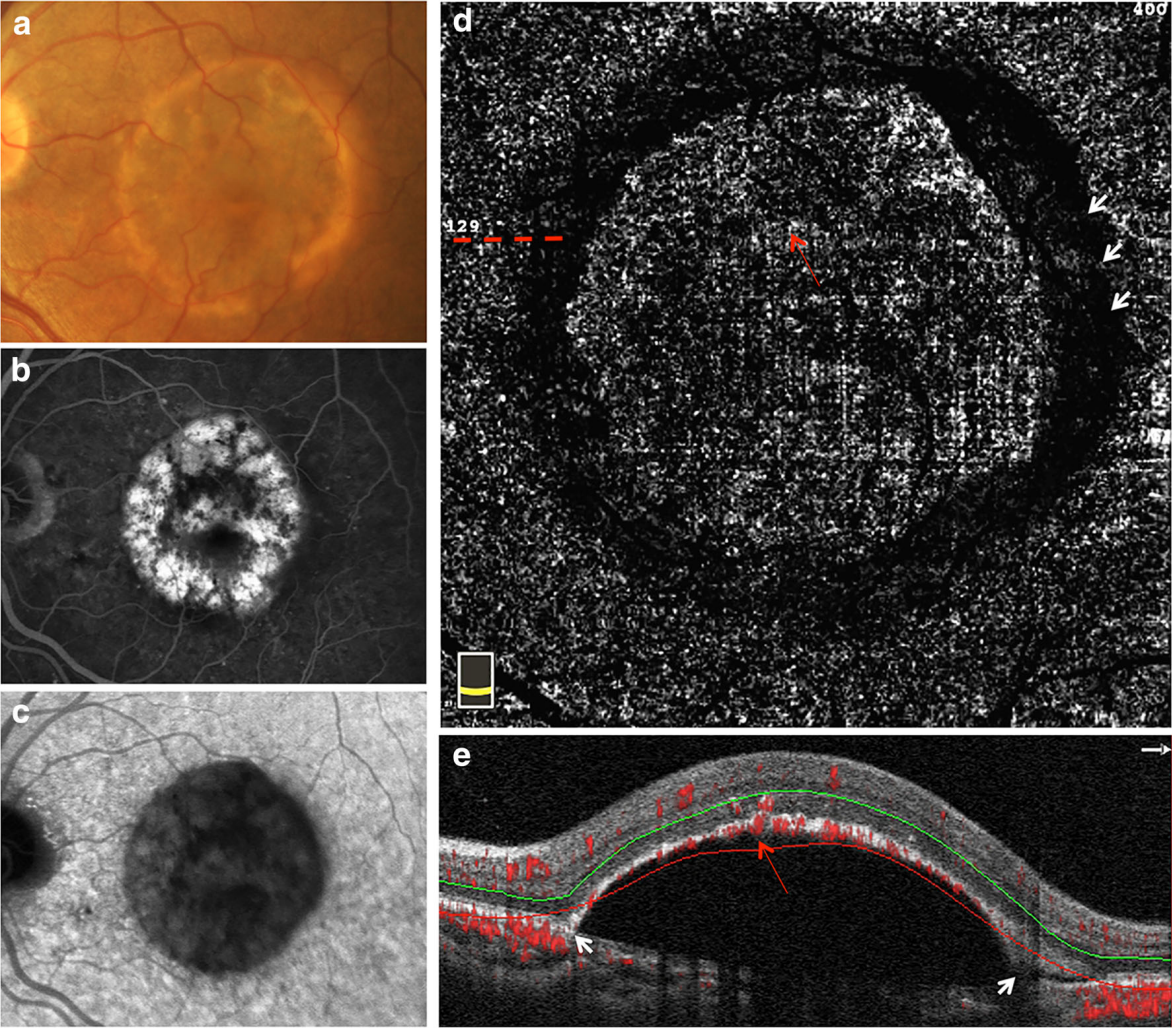
Multimodal imaging of non-vascularized PED in AMD with scattered RPE hyperplasia outside the central foveal avascular zone. A non-vascularized PED imaged on color fundus photography (**a**), late-phase fundus fluorescein angiography (**b**), late-phase indocyanine green angiography (**c**), the outer retina slab on en face OCTA (**d**), and cross-sectional OCTA with segmentation boundary lines (**e**). The dotted line shows the location of B-scans in **e**. The long red arrow shows the abnormal flow corresponding to the RPE hyperplasia overlying PED. The short white arrow in **d** shows the round-like dark band around the edge of PED. The short white arrow in **e** shows the steep edge of PED where the RPE was not segmented into the outer retina slab

In the present study, the VFS in the outer retina slab on OCTA was divided into three grades: (1), obvious VFS, vascular network visible; (2), VFS not obvious, blood flow fragments visible; and (3), no VFS. In addition, RPE hyperplasia was divided into four degrees: (1) lump RPE hyperplasia outside FAZ; (2) scattered RPE hyperplasia outside FAZ; (3) RPE hyperplasia inside FAZ; and (4) no RPE hyperplasia overlying PED. Table 1 shows the correspondence between RPE hyperplasia overlying PED and VFS at the outer retinal slab of OCTA. Findings showed that twelve eyes with lump RPE hyperplasia outside FAZ all showed obvious VFS at the outer retinal slab of OCTA, and 3 eyes with scattered RPE hyperplasia outside FAZ all showed blood flow fragments at the outer retinal slab of OCTA. Among them, 4 eyes had concurrent RPE hyperplasia inside FAZ, but it is worth noting that RPE hyperplasia inside FAZ all showed no VFS at the outer retinal slab of OCTA. Furthermore, 7 eyes had no RPE hyperplasia overlying PED, and all of them showed no VFS at the outer retinal slab of OCTA. In addition, a round-like dark band around the edge of PED was observed in the outer retina slab on en face OCTA in 17 eyes (77.3%) (Fig. 1g; Fig. 2d).

**Table 1:** The correspondence between RPE hyperplasia overlying PED and vascular flow signals in the outer retinal slab of OCTA

	VFS-vessels, *n* (%)	VFS-fragments, *n* (%)	No VFS, *n* (%)
Lump RPE-HP outside FAZ	12 (100%) (Fig. 1)	0	0
Scattered RPE-HP outside FAZ	0	3 (100%) (Fig. 2)	0
RPE-HP inside FAZ	0	0	4 (100%)* (Fig. 3)
No RPE-HP	0	0	7 (100%) (Fig. 4)

**Fig. 3:**
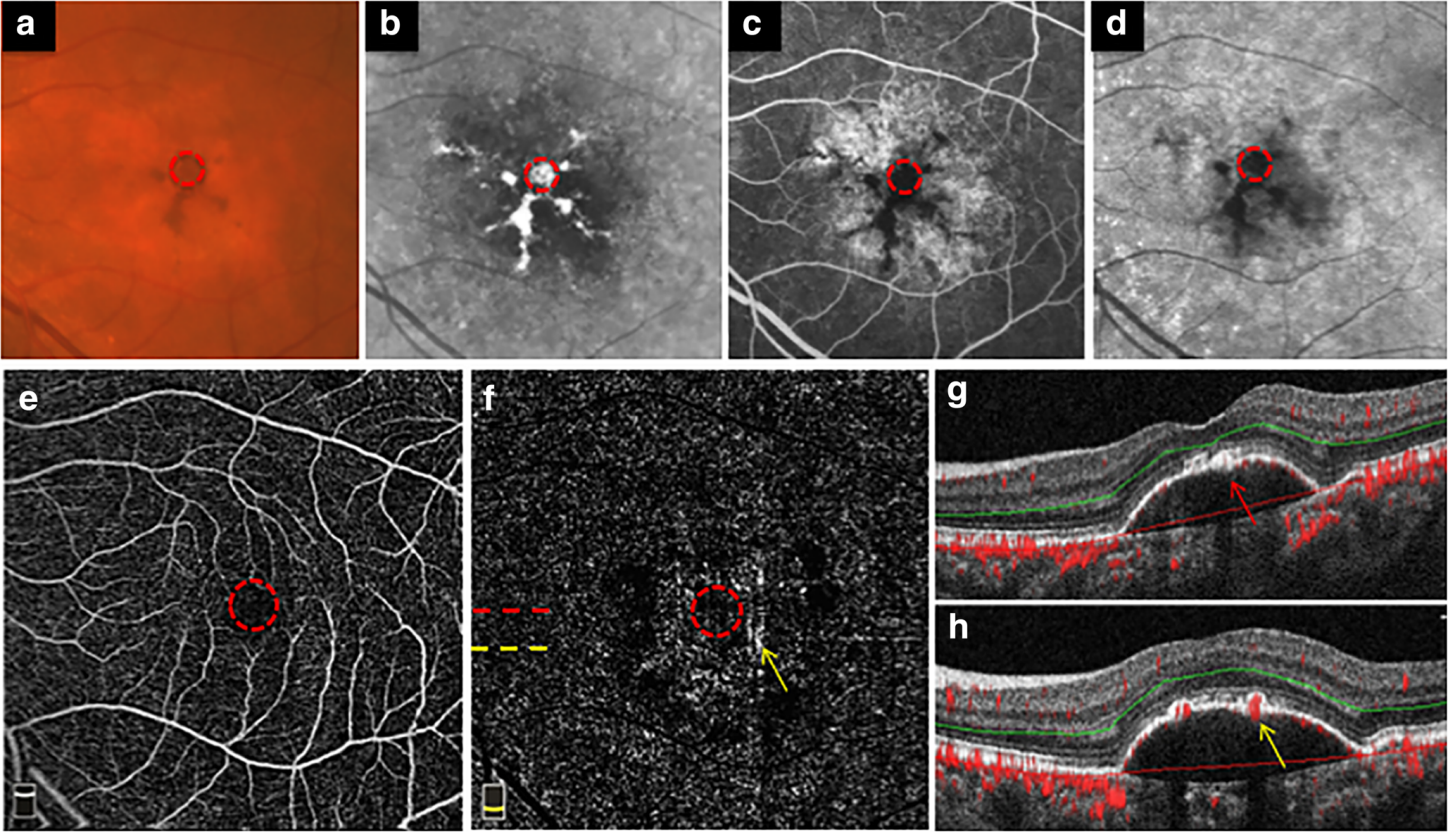
Multimodal imaging of non-vascularized PED in AMD with RPE hyperplasia inside and outside the central foveal avascular zone. A non-vascularized PED imaged on color fundus photography (**a**), infrared fundus photography (**b**), late-phase fundus fluorescein angiography (**c**), late-phase indocyanine green angiography (**d**), the superficial retinal slab on en face OCTA (**e**), the outer retina slab on en face OCTA (**f**), the cross-sectional OCTA through the foveal with segmentation boundary lines (**g**), and the cross-sectional OCTA outside the foveal with segmentation boundary lines (**h**). The dotted red circle in **a**–**f** shows the central foveal avascular zone (FAZ). The dotted red line in **f** shows the location of B-scan in **g**, which was through the foveal. The dotted yellow line in **f** shows the location of B-scan in **h**. The red arrow in **g** shows the RPE hyperplasia inside the central FAZ, with no abnormal vascular flow signal was shown. The yellow arrow in **f** and **h** shows the abnormal vascular flow corresponding to the RPE hyperplasia outside the central FAZ

**Fig. 4:**
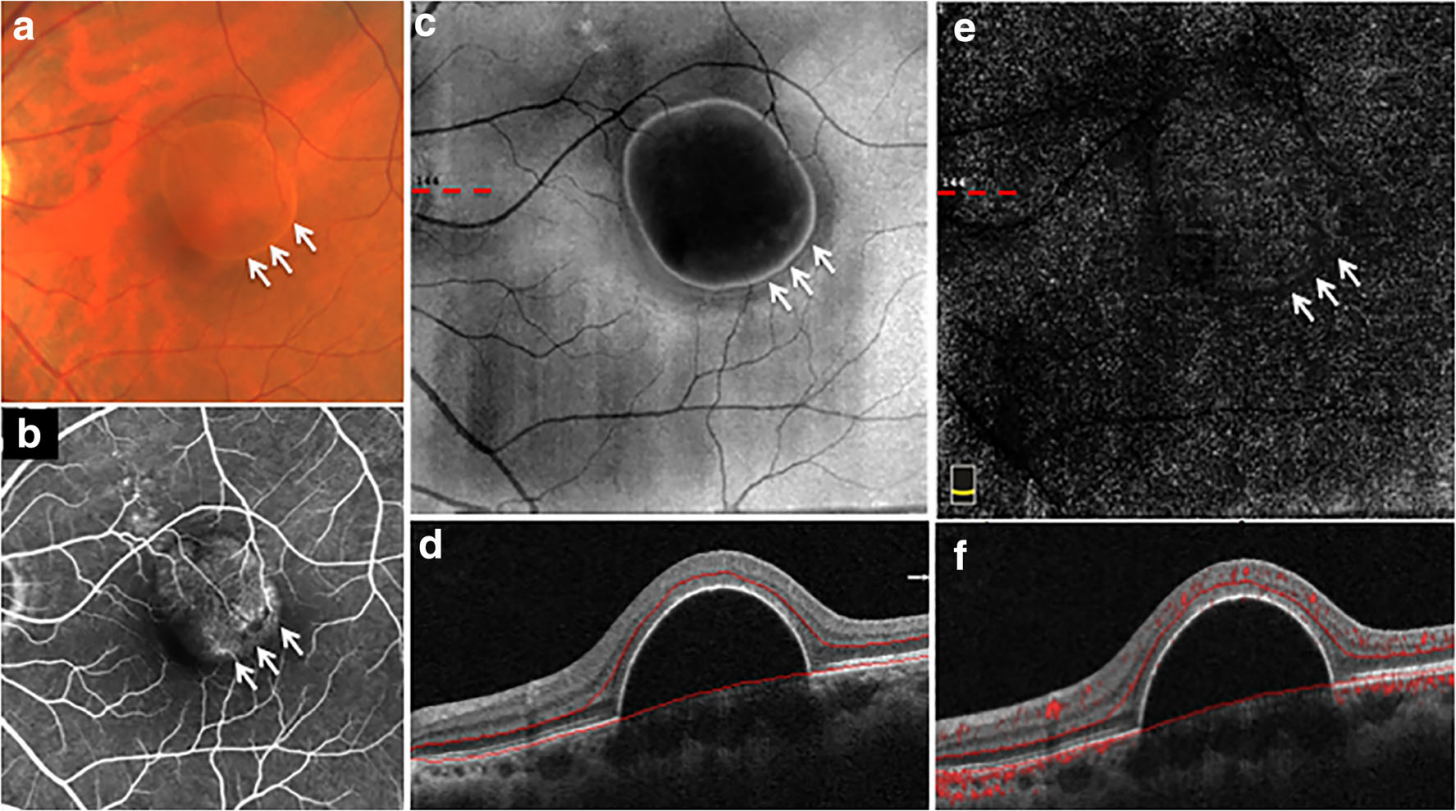
Non-vascularized PED in AMD without RPE hyperplasia and abnormal vascular flow signals on OCTA. A non-vascularized PED imaged on the color fundus photography (**a**), the late-phase fundus fluorescein angiography (**b**), the en face OCT (**c**), the cross-sectional OCT with segmentation boundary lines (**d**), the outer retina slab on en face OCTA (**e**), and the cross-sectional OCTA with segmentation boundary lines (**f**). The dotted line in **c** shows the location of B-scans in **d**. The dotted line in **e** shows the location of B-scans in **f**. The short white arrows in **a**, **b**, **c**, and **e** show the edge of PED. There is no RPE hyperplasia overlying PED inside and outside the central FAZ. And no vascular flow signal was observed at the outer retina slab of en face OCTA and the cross-sectional OCTA

Data represent *n* or %. *RPE*, retinal pigment epithelium; *PED*, pigment epithelial detachment; *OCTA*, optical coherence tomography angiography; *VFS*, vascular flow signals at the outer retinal slab of OCTA; *RPE-HP*, retinal pigment epithelium hyperplasia; *FAZ*, foveal avascular zone. *The four cases with RPE hyperplasia inside FAZ had concurrent RPE hyperplasia outside FAZ, and here we only focused on the VFS corresponding to the RPE hyperplasia inside FAZ. Figures 1–4 provided the standard photos for different degrees of RPE hyperplasia and VFS

## Non-vascularized PED in AMD with lump RPE hyperplasia outside FAZ

Figure 1 shows the multimodal imaging of represent example of non-vascularized PED in AMD with lump RPE hyperplasia outside FAZ. A round-like PED with RPE hyperplasia was located in the macular region. The RPE hyperplasia presented as yellow or brown lesions on color fundus photography (Fig. 1a), and hyper-autofluorescence on AF imaging (Fig. 1b). On FA, non-vascularized PED usually show faint hyperfluorescence in the early phase and increasingly pooling in the late stage. Focal hypofluorescence often corresponds to the blocking effect of overlying RPE hyperplasia (Fig. 1c). Using a confocal scanning laser ophthalmoscope (SLO) system, non-vascularized PEDs reveal hypofluorescence in both the early and the late phases of the ICGA [19, 20]. Lower fluorescence often corresponds to the blocking effect of RPE hyperplasia overlying PED (Fig. 1d). Moreover, RPE hyperplasia above PED appeared as hyperreflective lesions on IR image (Fig. 1e). On OCT, RPE hyperplasia presented as hyperreflective lesions with posterior shadowing (Fig. 1f). Of note, corresponding with the RPE hyperplasia overlying PED, obvious VFS in both the outer retina slab on en face OCTA (Fig. 1g) and the cross-sectional OCTA (Fig. 1h) were presented.

## Non-vascularized PED in AMD with scattered RPE hyperplasia outside FAZ

Figure 2 shows the multimodal imaging of represent example of non-vascularized PED in AMD with scattered RPE hyperplasia outside FAZ. A round-like PED with RPE hyperplasia was located in the macular region. The RPE hyperplasia showed scattered brown lesions on color FP (Fig. 2a), and corresponding blocked fluorescence on FA (Fig. 2b) and ICGA (Fig. 2c). In addition, corresponding with the RPE hyperplasia overlying PED, the blood flow fragments were observed in the outer retina slab on en face OCTA (Fig. 2d) as well as the cross-sectional OCTA (Fig. 2e).

## Non-vascularized PED in AMD with RPE hyperplasia inside and outside FAZ

Figure 3 shows the multimodal imaging of represent example of non-vascularized PED in AMD with RPE hyperplasia inside and outside FAZ. A round-like PED with RPE hyperplasia was located in the macular region. The RPE hyperplasia showed dark brown lesions on color fundus photography (Fig. 3a), hyperreflective lesions on IR image (Fig. 3b), and corresponding blocked fluorescence on FA (Fig. 3c) and ICGA (Fig. 3d). Multi-model images showed that the RPE hyperplasia located both inside and outside FAZ. The FAZ was indicated by red dotted circle in Fig. 3a–f. When the RPE hyperplasia located inside FAZ, there would be no VFS displayed in both the outer retina slab of en face OCTA (Fig. 3f) and the cross-sectional OCTA (Fig. 3g). Nevertheless, the RPE hyperplasia outside FAZ would have VFS in both the outer retina slab of en face OCTA (Fig. 3F) and the cross-sectional OCTA (Fig. 3h).

## Non-vascularized PED in AMD without RPE hyperplasia

Figure 4 shows the represent example of non-vascularized PED in AMD without RPE hyperplasia. A round-like PED in the macular region was shown in color fundus photography (Fig. 4a) and fundus fluorescence angiography (Fig. 4b), without neovascularization and RPE hyperplasia. The en face OCT (Fig. 4c) and cross-sectional OCT (Fig. 4d) showed the PED clearer. In addition, there was no VFS displayed in both the outer retina slab of en face OCTA (Fig. 4e) and the cross-sectional OCTA (Fig. 4f).

## Discussion

With OCTA technology being increasingly used, various image artifacts in OCTA were observed and reported [8–11]. Their origin, recognition, interpretation, and avoidance are crucial to prevent possible misdiagnosis. In the present study, we reported the image artifacts due to RPE hyperplasia overlying non-vascularized PED in AMD, which are very common and can masquerade as neovascularization on OCTA.

OCTA is a functional extension of structural OCT, which performs repeated B-scans to detect motion contrast and visualize vasculature of retina and choroid. In brief, repeated cross-sectional scans are captured at the same retinal location and in rapid sequence. The relative change of signal is measured at each voxel, and flow is considered present if the change reaches a determined threshold [21, 22]. OCTA has the advantage that can visualize microvasculature with depth resolution. In contrast to FA or ICGA, OCTA images are not obscured by hyperfluorescence from dye leakage. Since OCTA does not require administration of exogenous contrast, it can be performed at any patient visit. However, various image artifacts, including motion, fringe washout, decorrelation projection, masking, unmasking, and stromal decorrelation signal were reported [11]. Moreover, artifacts includes segmentation, motion, blink, masking, unmasking, vessel doubling, and banding and out of window artifacts were demonstrated common in OCTA images, especially in eyes with pathology [10]. Furthermore, study showed that the anterior displacement of larger choroidal vessels in geographic atrophy could masquerade as choroidal neovascularization (CNV) on en face OCTA, so cross-sectional OCTA used in conjunction with en face OCTA was necessary for confirming the presence of CNV [18, 23].

Our study showed that VFS was observed in both the outer retinal slab of en face OCTA and the cross-sectional OCTA in 15 of 22 included eyes, and all of them had corresponding RPE hyperplasia overlying PED. This VFS was considered to be image artifacts since only non-vascularized PED, according to multi-model imaging, were included in this study. Previous study showed that image artifacts could arise from the OCT image acquisition, intrinsic characteristics of the eye, eye motion, or image processing and display strategies [8]. Most of image artifacts in OCTA were easy to identify and might be reduced with the real-time tracking and motion correction technology [24]. Furthermore, projection artifacts from the overlying retinal circulation could be automatically removed from the outer retinal slab on en face OCTA with a projection artifact removal algorithm [25]. However, although using the eye tracking system and the projection artifact removal algorithm, we still observed VFS on the outer retinal slab of OCTA. Through the comparison with other multimodal imaging including IR, FA, ICGA, and OCT, we found that the position and pattern of VFS on OCTA were corresponding to the position and pattern of RPE hyperplasia overlying PED exactly, except for the RPE hyperplasia located inside the FAZ. So, this RPE hyperplasia-related image artifacts might be associated with the overlying retinal circulation. Previous study demonstrated histologically that a vascular complex implanted into sub-RPE basal laminar deposit was characteristic of type 3 neovascularization which was closely related to a mixed serous and drusenoid PED [14]. Therefore, the RPE hyperplasia-related image artifacts are easily misunderstood as type 3 neovascularization on OCTA in clinical.

Then, how did this OCTA image artifacts produce? As we know, backscattered light from structures directly underneath blood vessels can exhibit false decorrelation as it passes through a more superficial blood vessel layer on its way back to the OCT detector. This explains how the decorrelation projection artifacts produce and why it almost always presents in any structure located below vessels. RPE, as a highly reflective layer, the overlying retinal blood vessels are projected onto it to produce marked decorrelation projection artifacts. Fortunately, a projection artifact removal algorithm was available to remove the decorrelation projection artifacts automatically. However, the RPE hyperplasia overlying PED would increase the intensity of backscattered light locally, so the decorrelation projection artifacts were augmented locally. And the augmented part of projection artifacts due to RPE hyperplasia could not be removed, so VFS was presented there. Furthermore, our study showed that the pattern of VFS on OCTA was corresponded to the pattern of RPE hyperplasia outside the FAZ. Nevertheless, no VFS was observed on OCTA when RPE hyperplasia located inside the FAZ, further confirmation of our inferences. There were no retinal blood vessels overlying RPE inside the FAZ, so no projection artifacts caused by RPE hyperplasia there. Understanding this RPE hyperplasia-related OCTA, image artifacts were very important for reducing the risk of misdiagnosis.

In addition, our study showed that a round-like dark band around the edge of PED was observed in the outer retina slab on en face OCTA in 17 cases. We have explored the cause of the dark band on OCTA very carefully by a dynamic OCTA analysis. And we found that the dark band was corresponding to the segmentation errors on OCTA B-scans at the steep edge of PED, where the RPE was not segmented into the outer retina slab, so no projection artifact caused by RPE there. But the projection artifact removal algorithm was commonly used to remove the projection artifacts in the outer retina slab by subtraction. Therefore, we speculated that the round-like dark band might be a consequence of both the segmentation errors at the steep edge of PED and the projection artifact removal algorithm application.

There are several limitations to our study that need to be considered. Firstly, as a clinic-based cross-sectional observational study, selection bias may exist. Secondly, we included a small number of eyes in this study. Thirdly, all patients were from a single institution, so a referral bias may exist. However, to the best of our knowledge, this is the first study to investigate the OCTA features of non-vascularized PED in AMD and to describe OCTA image artifacts due to RPE hyperplasia overlying PED. Of note, this image artifact represents amplified projection artifact and is easy to be misdiagnosed as type 3 neovascularization secondary to AMD.

In conclusion, RPE hyperplasia overlying PED in AMD can masquerade as neovascularization on OCTA, which might be a kind of augmented decorrelation projection artifacts. To avoid misdiagnosis and unnecessary treatment, this RPE hyperplasia-related image artifacts should be considered when interpreting OCTA images.

## References

[1] Makita S, Hong Y, Yamanari M, Yatagai T, Yasuno Y (2006). Optical coherence angiography. Opt Express.

[2] Matsunaga D, Yi J, Puliafito CA, Kashani AH (2014). OCT angiography in healthy human subjects. Ophthalmic surgery, lasers & imaging retina.

[3] Samara WA, Shahlaee A, Sridhar J, Khan MA, Ho AC, Hsu J (2016). Quantitative optical coherence tomography angiography features and visual function in eyes with branch retinal vein occlusion. Am J Ophthalmol.

[4] Runkle AP, Kaiser PK, Srivastava SK, Schachat AP, Reese JL, Ehlers JP (2017). OCT angiography and ellipsoid zone mapping of macular telangiectasia type 2 from the AVATAR study. Invest Ophthalmol Vis Sci.

[5] Ahmed Daniel, Stattin Martin, Graf Alexandra, Forster Julia, Glittenberg Carl, Krebs Ilse, Ansari-Shahrezaei Siamak (2018). DETECTION OF TREATMENT-NAIVE CHOROIDAL NEOVASCULARIZATION IN AGE-RELATED MACULAR DEGENERATION BY SWEPT SOURCE OPTICAL COHERENCE TOMOGRAPHY ANGIOGRAPHY. Retina.

[6] Bonini Filho MA, de Carlo TE, Ferrara D, Adhi M, Baumal CR, Witkin AJ, Reichel E, Duker JS, Waheed NK (2015). Association of choroidal neovascularization and central serous chorioretinopathy with optical coherence tomography angiography. JAMA ophthalmology.

[7] Lu Y, Simonett JM, Wang J, Zhang M, Hwang T, Hagag AM, Huang D, Li D, Jia Y (2018). Evaluation of automatically quantified foveal avascular zone metrics for diagnosis of diabetic retinopathy using optical coherence tomography angiography. Invest Ophthalmol Vis Sci.

[8] Spaide RF, Fujimoto JG, Waheed NK (2015). Image artifacts in optical coherence tomography angiography. Retina.

[9] Ferrara D (2016). Image artifacts in optical coherence tomography angiography. Clin Exp Ophthalmol.

[10] Ghasemi Falavarjani K, Al-Sheikh M, Akil H, Sadda SR (2017). Image artefacts in swept-source optical coherence tomography angiography. Br J Ophthalmol.

[11] Chen FK, Viljoen RD, Bukowska DM (2016). Classification of image artefacts in optical coherence tomography angiography of the choroid in macular diseases. Clin Exp Ophthalmol.

[12] Mrejen S, Sarraf D, Mukkamala SK, Freund KB (2013). Multimodal imaging of pigment epithelial detachment: a guide to evaluation. Retina.

[13] Tan Anna C. S., Freund K. Bailey, Balaratnasingam Chandrakumar, Simhaee Daniel, Yannuzzi Lawrence A. (2018). IMAGING OF PIGMENT EPITHELIAL DETACHMENTS WITH OPTICAL COHERENCE TOMOGRAPHY ANGIOGRAPHY. Retina.

[14] Li M, Dolz-Marco R, Messinger JD, Wang L, Feist RM, Girkin CA, Gattoussi S, Ferrara D, Curcio CA, Freund KB (2018). Clinicopathologic correlation of anti-vascular endothelial growth factor-treated type 3 neovascularization in age-related macular degeneration. Ophthalmology.

[15] Alten F, Lauermann JL, Clemens CR, Heiduschka P, Eter N (2017). Signal reduction in choriocapillaris and segmentation errors in spectral domain OCT angiography caused by soft drusen. Graefe’s archive for clinical and experimental ophthalmology = Albrecht von Graefes Archiv fur klinische und experimentelle Ophthalmologie.

[16] Cukras C, Agron E, Klein ML, Ferris FL, Chew EY, Gensler G, Wong WT, Age-Related Eye Disease Study Research G (2010). Natural history of drusenoid pigment epithelial detachment in age-related macular degeneration: age-related eye disease study report no. 28. Ophthalmology.

[17] Jia YL, Tan O, Tokayer J, Potsaid B, Wang YM, Liu JJ, Kraus MF, Subhash H, Fujimoto JG, Hornegger J, Huang D (2012). Split-spectrum amplitude-decorrelation angiography with optical coherence tomography. Opt Express.

[18] Kvanta A, Casselholm de Salles M, Amren U, Bartuma H (2017). Optical coherence tomography angiography of the foveal microvasculature in geographic atrophy. Retina.

[19] Arnold JJ, Quaranta M, Soubrane G, Sarks SH, Coscas G (1997). Indocyanine green angiography of drusen. Am J Ophthalmol.

[20] Flower RW, Csaky KG, Murphy RP (1998). Disparity between fundus camera and scanning laser ophthalmoscope indocyanine green imaging of retinal pigment epithelium detachments. Retina.

[21] Kim DY, Fingler J, Werner JS, Schwartz DM, Fraser SE, Zawadzki RJ (2011). In vivo volumetric imaging of human retinal circulation with phase-variance optical coherence tomography. Biomedical optics express.

[22] Jia Y, Tan O, Tokayer J, Potsaid B, Wang Y, Liu JJ, Kraus MF, Subhash H, Fujimoto JG, Hornegger J, Huang D (2012). Split-spectrum amplitude-decorrelation angiography with optical coherence tomography. Opt Express.

[23] Nesper PL, Lutty GA, Fawzi AA (2018). Residual choroidal vessels in athrophy can masquerade as choroidal neovascularization on optical coherence tomography angiography: introducing a clinical and software approach. Retina.

[24] Camino A, Zhang M, Gao SS, Hwang TS, Sharma U, Wilson DJ, Huang D, Jia Y (2016). Evaluation of artifact reduction in optical coherence tomography angiography with real-time tracking and motion correction technology. Biomedical optics express.

[25] Zhang Q, Zhang A, Lee CS, Lee AY, Rezaei KA, Roisman L, Miller A, Zheng F, Gregori G, Durbin MK, An L, Stetson PF, Rosenfeld PJ, Wang RK (2017). Projection artifact removal improves visualization and quantitation of macular neovascularization imaged by optical coherence tomography angiography. Ophthalmology retina.

